# Protective effects of *Paeonia suffruticosa* callus extract in skin through anti‐inflammation and repair UVB‐induced damage

**DOI:** 10.1111/ics.13055

**Published:** 2025-03-13

**Authors:** Yufan Li, Jiejun Han, Rongyue Gong, Yuankun Liu, Yu Zhou, Tiangui Gong, Bin Wang, Laidi Zhang, Shuodan Li, Jiayue Chen

**Affiliations:** ^1^ State Key Laboratory of Protein and Plant Gene Research, School of Advanced Agriculture Sciences and School of Life Sciences Peking University Beijing China; ^2^ Hangzhou Shiguang Xinya Biotechnology Co., Ltd. Hangzhou China; ^3^ MCL Skincare Ltd. Hangzhou China

## Abstract

**Objective:**

The study investigated effects of peony callus extracts (PCE) on the protective efficacy against Ultraviolet B (UVB)‐induced photoageing, using in vitro and in vivo studies. The research focused on PCE's ability to protect against inflammatory factors, DNA damage and accumulation of senescent cells, along with the evaluation of the extract's potential anti‐photoageing benefits to skin.

**Methods:**

Human keratinocyte cell line (HaCaT cells), mast cells and fibroblasts were used to evaluate the role of PCE in anti‐photoageing. The expression of genes of *interleukin‐1α (IL‐1α)*, *IL‐6* and transient receptor potential vanilloid 1 (*TRPV1*) were tested in HaCaT cells. The histamine contents in mast cells were tested to evaluate the effect of PCE on soothing skin. Additionally, the repairment of PCE on DNA damage stimulated by UVB using comet assay was evaluated. In fibroblasts, the gene expression of *matrix metalloproteinases* (*MMPs*) and the activity of β‐galactosidase were tested. In vivo test, 13 healthy volunteers were enrolled to apply a formula with 1% PCE to assess the variation in inner skin collagen contents.

**Results:**

The callus from an ancient and rare variety of tree peony (*Paeoniaceae* family) was successfully induced, and its ingredients were extracted. The PCE could significantly downregulate inflammation factors such as *IL‐1α*, *IL‐6* and *TRPV1* in HaCaT cells, and *MMPs* in fibroblasts which could cause the collagen degradation induced by UVB. Meanwhile, UVB‐induced DNA damage was alleviated by PCE. The analysis of histamine content in mast cells revealed that PCE effectively alleviated skin sensitivity. Furthermore, the clinical trials validated a significant increase in total collagen content in vivo, following 28 days of continuous application of a cosmetic formulation containing 1% PCE measured by Raman confocal spectroscopy technology.

**Conclusion:**

The PCE could downregulate the gene expression of inflammatory factors, indicating the ability of DNA repair. The number of senescent cells was also decreased after UVB stimulation. Furthermore, the results of in vivo study showed that PCE was an ideal cosmetic ingredient for promoting collagen levels.

## Graphical abstract

Peony callus extract (PCE) is beneficial for skin anti‐ageing and sensitivity induced by UVB radiation, through decreasing the level of inflammatory factors and matrix metalloproteinases. Furthermore, our research indicates that PCE effectively improves the total collagen content in human skin.

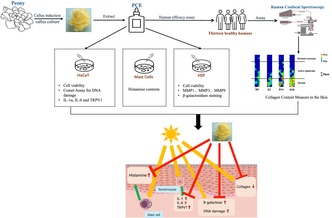

## Résumé

**Objectif:**

L'étude a examiné les effets des extraits de cal de pivoine (PCE) sur l'efficacité protectrice contre le photo‐vieillissement induit par les UVB (Ultraviolet B), en utilisant des études in vitro et in vivo. La recherche s'est concentrée sur la capacite des PCE a protéger contre les facteurs inflammatoires, les dommages à l'ADN et l'accumulation de cellules sénescentes, ainsi que sur l'évaluation des bénéfices potentiels anti‐photo‐ vieillissement de l'extrait pour la peau.

**Méthodes:**

Une lignée cellulaire de keratinocytes humains (cellules HaCaT), des mastocytes et des fibroblastes ont été utilisés pour évaluer le rôle des PCE dans l'anti‐photo‐ vieillissement. L'expression des gènes de l'interleukine‐1α (IL‐1α), de l'IL‐6 et du récepteur transitoire potentiel vanilloide 1 (TRPV1) a été testée dans les cellules HaCaT. Les contenus en histamine des mastocytes ont été testés pour évaluer l'effet des PCE sur l'apaisement de la peau. De plus, la réparation des dommages a l'ADN stimules par les UVB par les PCE a été évaluée à l'aide du test de comète. Dans les fibroblastes, l'expression des gènes des métalloprotéinases matricielles (MMPs) et l'activité de la β‐galactosidase ont été testées. Dans le test in vivo, 13 volontaires sains ont été recrutes pour appliquer une formule contenant 1% de PCE afin d'évaluer la variation des contenus en collagène interne de la peau.

**Résultats:**

Le cal d'une variété ancienne et rare de pivoine (famille des Paeoniaceae) a été induit avec succès, et ses ingrédients ont été extraits. Les PCE ont pu significativement réduire l'expression des facteurs inflammatoires tels que l'IL‐1α, l'IL‐6 et le TRPV1 dans les cellules HaCaT, ainsi que les MMPs dans les fibroblastes, qui peuvent causer la dégradation du collagène induite par les UVB. Parallèlement, les dommages à l'ADN induits par les UVB ont été attenues par les PCE. L'analyse du contenu en histamine dans les mastocytes a révélé que les PCE ont efficacement apaise la sensibilité de la peau. De plus, les essais cliniques ont validé une augmentation significative du contenu total en collagène in vivo, après 28 jours d'application continue d'une formulation cosmétique contenant 1% de PCE, mesurée par la technologie de spectroscopie confocale Raman.

**Conclusion:**

Les PCE ont pu réduire l'expression des gènes des facteurs inflammatoires, indiquant une capacite de réparation de l'ADN. Le nombre de cellules sénescentes a également diminue après stimulation par les UVB. En outre, les résultats de l'étude in vivo ont montré que les PCE constituent un ingrédient cosmétique idéal pour promouvoir les niveaux de collagène.

## INTRODUCTION

*Paeonia Suffruticosa* (tree peony) is a flower that belongs to the *Paeoniaceae* family and *Paeonia* genus. The flower's origins trace back to China, where it grows in abundance. The tree peony extract has great medicinal value emanating from its rich phytochemical composition [1, 2, 3]. In Europe, practitioners have used the flower in various forms of traditional Chinese medicine (TCM) and traditional herbalism to capitalize on its therapeutic properties. There is a 400‐year‐old ‘Peony King’ in China, known as ‘Yucui Hehua’, which can be traced back to the Ming Dynasty (1610; https://lhsr.sh.gov.cn/). As of today, Yucui Hehua is cultured at Baihua Garden in Weifang City, Shandong Province and Shanghai. In recent years, more people have used botanical ingredients. For example, in cosmeceuticals, manufacturers use tree peony extracts which offer anti‐inflammatory and antioxidant benefits to consumers [4]. Thus, elder peony is prevalent in the cosmetics field because of its potential efficacy.

Intrinsic and extrinsic factors influence the complex process of skin ageing. Classically, intrinsic ageing is primarily driven by genetically determined chronological factors and physiological changes. On the other hand, the researchers argue that ultraviolet (UV) radiation, air pollutants and other environmental factors mainly cause extrinsic ageing [5, 6]. During skin ageing, the skin structure and function deteriorate. After deteriorating, the skin changes in appearance developing wrinkles, sagging, pigmentation spots, dryness and irregular skin texture [7]. Numerous studies that focused on extrinsic factors concluded that skin ageing mainly comes from exposure to UVB (short‐wave UVB, 280‐320 nm) radiation [8, 9]. The skin produces reactive oxygen species (ROS) when it is exposed to UVB. Accumulation of ROS causes oxidative stress and damages subcellular structures such as DNA, proteins and lipids [10]. Subsequently, skin cells function abnormally and a series of reactions in molecular events that contribute to ageing are initiated because of damaged subcellular structures. Therefore, understanding the mechanisms by which UVB radiation induces skin ageing is essential for developing effective strategies to protect against premature ageing and maintain skin health.

The release of inflammatory cytokines or other mediators in the skin, such as interleukin‐1 (IL‐1), IL‐6 and tumour necrosis factor‐alpha (TNF‐α), is triggered by exposure of the skin to UVB [11, 12]. Simultaneously, the inflammatory factors are a catalyst that activates called matrix metalloproteinases (MMPs). MMPs degrade collagen and elastin fibres in the extracellular matrix of the skin [12, 13]. Consequently, when more MMPs are activated, the skin's structural components decompose resulting in wrinkle formation and loss of skin elasticity [10]. In this study, we explored the complex relationship between UVB radiation and skin ageing. The study mainly focused on the molecular pathways involved and discussed some potential ways to mitigate damage from UVB radiation exposure and preserve youthful skin appearance.

Raman confocal spectroscopy is analytical machine commonly used in vivo assay. The analytical machine is superior for its non‐invasive, real‐time, in situ and multi‐component detection. Raman confocal spectroscopy has vast potential for use in the field of skin penetration [14, 15]. Lee et al. [16] and Lintzeri et al. [17] studied a way to distinguish the skin layers. In the process, they discovered a method that uses Raman spectroscopy in vivo. A depth two‐dimensional imaging using the analytical machine could produce good results for exogenous actives that promote changes in endogenous biomolecules such as collagen in the skin [18]. We monitored the changes in endogenous collagen levels in human skin to detect the effect of endogenous collagen by PCE.

## RESULTS

### Peony callus culture and main ingredient measurement

**FIGURE 1:**
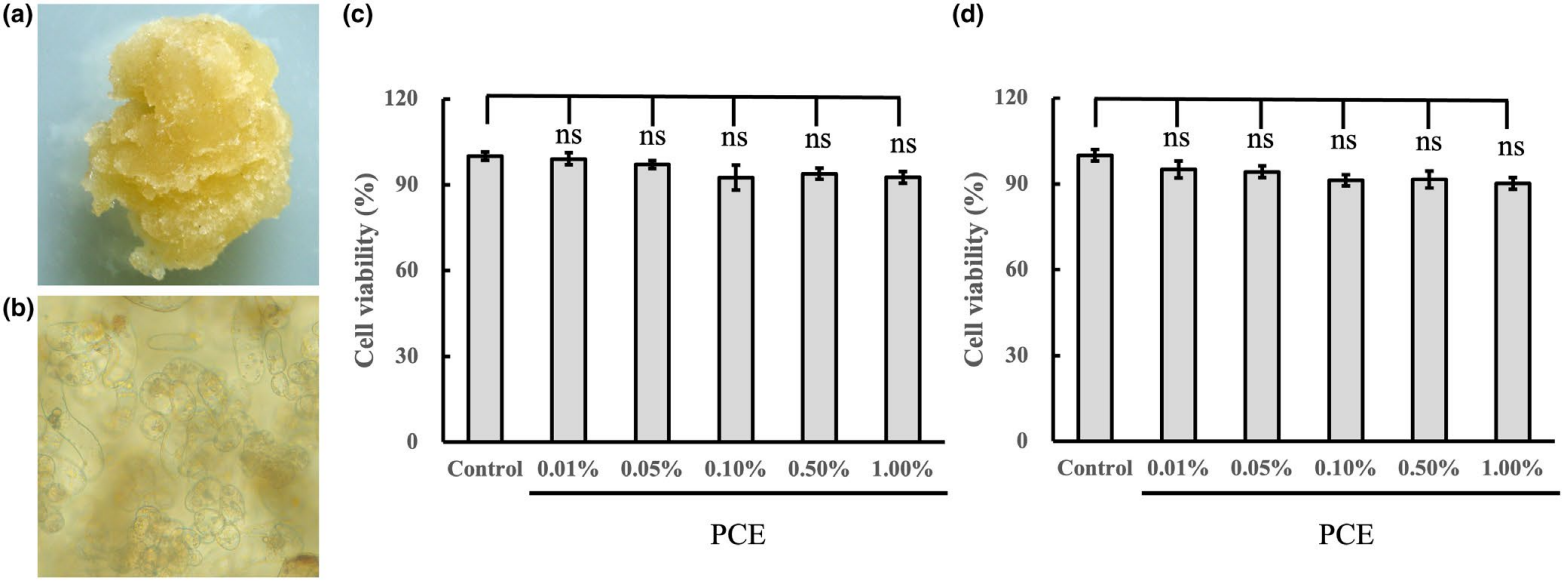
The morphology of PCE callus and its cytotoxicity to HaCaT and HSF cells. (a) Fresh morphology of callus of peony. (b) Microscopy images of peony callus cells. (c) Effects of PCE at different concentrations (0.01%, 0.05%, 0.1%, 0.5% and 1%) on the cell viability in HaCaT cells. (d) Effects of PCE at different concentrations (0.01%, 0.05%, 0.1%, 0.5% and 1%) on the cell viability in HSF cells. PCE represents peony callus extract.

The study used peony buds as explants to induce callus. Initially, a culturing process of explants on an MS medium occurred to obtain a callus. Subsequently, we tested the effects of different hormones on callus growth (data not shown). The results showed that MS medium supplemented with 30 g L^−1^ of sucrose, 7.5 g L^−1^ of agar powder, 0.5 mg L^−1^ 6‐BA, 0.5 mg L^−1^ NAA and 1 mg L^−1^ TDZ is the optimal medium for peony callus culture. Figure 1a shows a light‐yellow peony callus obtained 25 days later after culturing. Using a microscope, the cells of callus could be clearly observed (Figure 1b). We performed HPLC to determine the relative portion of paeoniflorin and paeonol (Figure S1) which helped us identify the main components in the PCE. Paeoniflorin and paeonol (Figure S1) are considered the main pharmacological actives in peony extract [19]. In peony callus extract (PCE), we found 0.29 ± 0.02 mg mL^−1^ and 0.22 ± 0.01 mg mL^−1^ levels of paeoniflorin and paeonol.

### Effects of PCE on cell viability

We evaluated the efficacy of PCE. The first step involved measuring the viability of both human keratinocytes cell line (HaCaT) and fibroblasts treated with different concentrations (0.01%, 0.05%, 0.1%, 0.5% and 1%) of PCE. We observed no significance among the blank control and PCE treatments at 0.01%–1%, cell viability remained above 92% in HaCaT cells (*p* > 0.05; Figure 1c) and 90% in fibroblasts (*p* > 0.05; Figure 1d) among the tested concentrations. The findings showed that PCE at concentrations of 0.01%–1% did not exhibit apparent cytotoxicity towards both HaCaT and fibroblasts. The experiments that followed later used the PCE concentrations of 0.1%, 0.5% and 1%.

### Evaluation of the skin‐soothing effects of PCE

**FIGURE 2:**
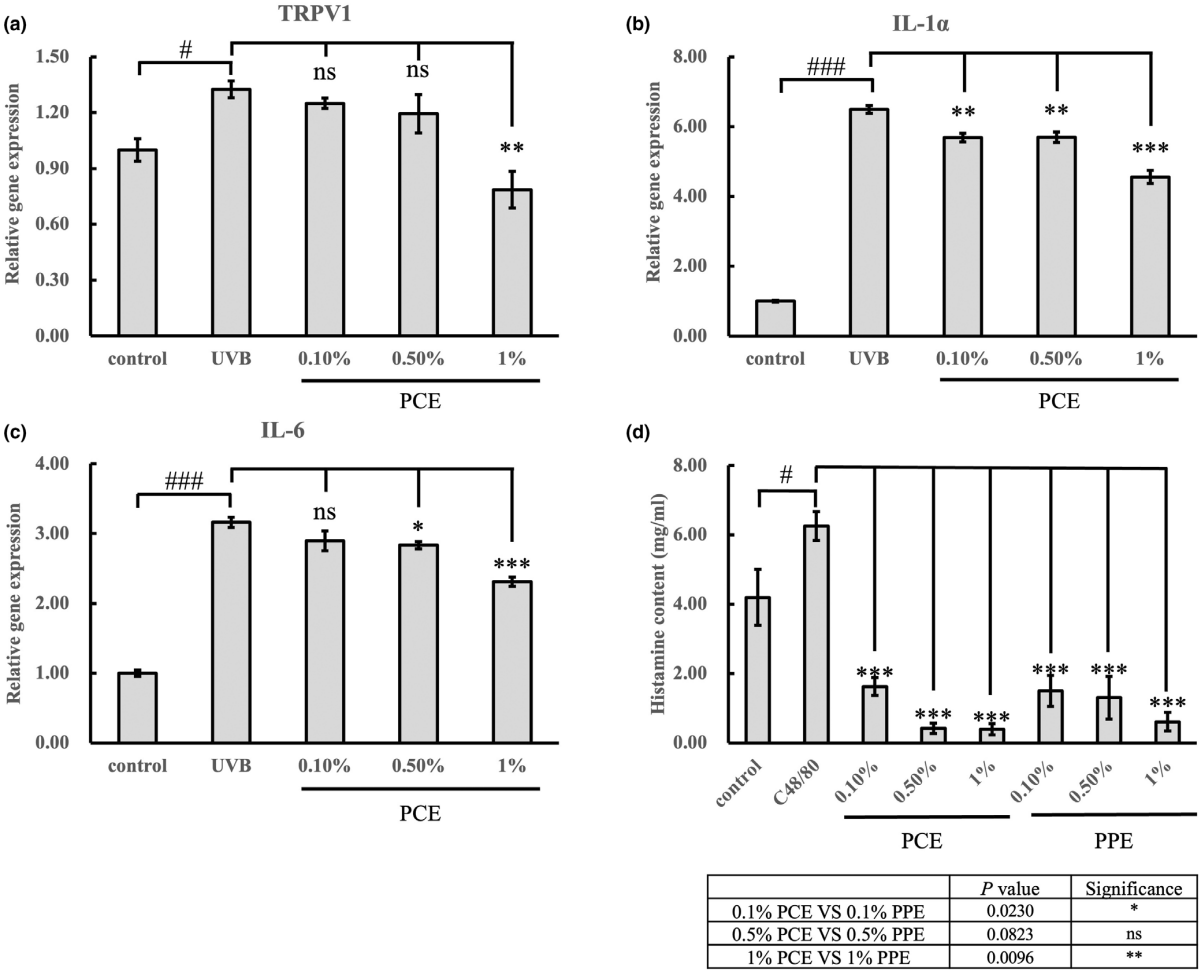
Related evaluation of the soothing effects of PCE in HaCaT cells and mast cells. The relative gene expression of TRPV1 (a), IL‐1α (b) and IL‐6 (c) in HaCaT cells. (d) The histamine levels in mast cells. One‐way analysis of variance (ANOVA) was performed under model conditions. *p < 0.05; **p < 0.01; ***p < 0.001; n.s. means no significance (*n* = 3). The Student's *t*‐test was compared between control and model groups (UVB exposure, C48/80 treatment). #*p* < 0.05; ###*p* < 0.001 (*n* = 3).

To assess the soothing effect, we explored alterations in the expression levels of *TRPV1* (transient receptor potential vanilloid 1), *IL‐1α* and *IL‐6* in HaCaT cells after exposure to UVB, along with the release of histamine in mast cells. Compared to blank control, UVB radiation significantly elevated the expression level of *TRPV1*, *IL‐1α* and *IL‐6* by 40.8%, 29.8% and 26.9%, respectively (*p* < 0.01; Figure 2a–2c). When the concentration of PCE exceeded 0.5%, compared to the UVB treatment, PCE downregulates the expression of *TRPV1* which is expressed in epidermal nerve endings and is intricately involved in mediating skin sensitivity to various stimuli. Specifically, 0.5% (*p* < 0.05) and 1% (*p* < 0.01). PCE significantly reduced the gene expression of *TRPV1* by 10.0% and 40.8%, respectively (Figure 2a). In addition, PCE at concentrations of both 0.5% and 1% (*p* < 0.05; Figure 2b,c), eliminated the presence of common pro‐inflammatory cytokines *IL‐1α* and *IL‐6* induced by UVB.

Histamine is a common inflammatory mediator. The inflammation is used to treat acute allergic responses and modulate chronic inflammation and immune homeostasis. We tested histamine content within C48/80‐induced mast cells to interpret the impact of PCE on histamine release. Researchers have widely used this model to study histamine‐related physiological and pathological responses, such as inflammation, allergic reactions and drug screening. Statistical analysis from the blank control revealed an eminent increase in histamine by C48/80 induction. However, there is a significant reduction in histamine when treated with PCE, even at the concentration of 0.1% (73.9% reduction; *p* < 0.01; Figure 2d). Meanwhile, 0.5% and 1% PCE treatments showed a stronger ability to reduce histamine release compared to PPE (peony plant extract) treatments at the same concentration (Figure 2d). From these results, it could be seen that soothing effects of PCE are significant after exposure to UVB radiation.

### PCE facilitated the repairment of UVB‐induced DNA damage

**FIGURE 3:**
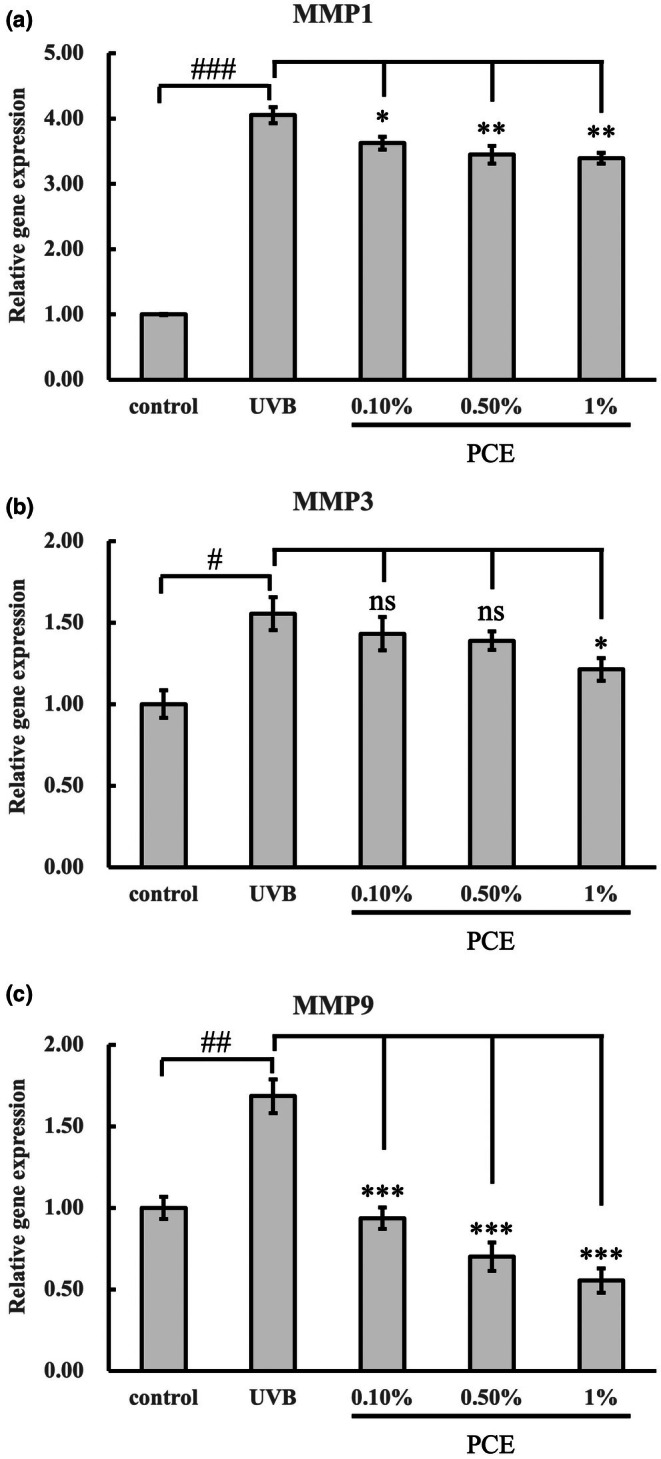
Inhibitory effect of PCE on MMP1 (a), MMP3 (b) and MMP9 (c) to HSF cells stimulated by UVB. One‐way analysis of variance (ANOVA) was performed under UVB condition. *p < 0.05; **p < 0.01; ***p < 0.001 (*n* = 3); n.s. means no significance. The Student's *t*‐test was compared between control and UVB treatment. #*p* < 0.05; ##*p* < 0.01; ###*p* < 0.001 (*n* = 3).

DNA damage is the main cause of cell ageing. Therefore, proper care of DNA integrity is vital for skin anti‐photoageing. When the DNA is damaged, it ejects from the cell nucleus and forms a DNA tail that resembles a comet [20]. We assessed UVB‐induced DNA damage and investigated how PCE protects against UVB. The assessment involved running an alkaline assay on the DNA tail. UVB significantly increased the tail length by 15.8 times, DNA tail content by 11.6 times and DNA moment by 26.0 times (*p* < 0.001; Figure 3) compared to blank control. This unveiled that UVB exposure substantially induced DNA damage in HaCaT cells. Nonetheless, adding PCE, even at a minimal concentration of 0.1%, significantly reduced UVB‐induced DNA damage. The results showed an eminent effect of PCE on reducing tail length (310.73 ± 73.6 vs. 426.73 ± 82.9; *p* < 0.001), decreasing the percentage of tail DNA (36.54 ± 12.9 vs. 66.14 ± 14.7, *p* < 0.001) and lowering DNA moment (80.87 ± 24.1 vs. 145.78 ± 36.1, *p* < 0.001), compared to the blank control (0.01% PCE vs. blank control; Figure 3). In parallel, the role of 1% PCE on DNA protection was stronger than PPE, consistent with its greater reduction in tail DNA content and decrease in tail length (Figure 3). It suggested the potential efficacy of PCE in protecting from UVB.

### PCE modulated the gene expressions of MMPs

**FIGURE 4:**
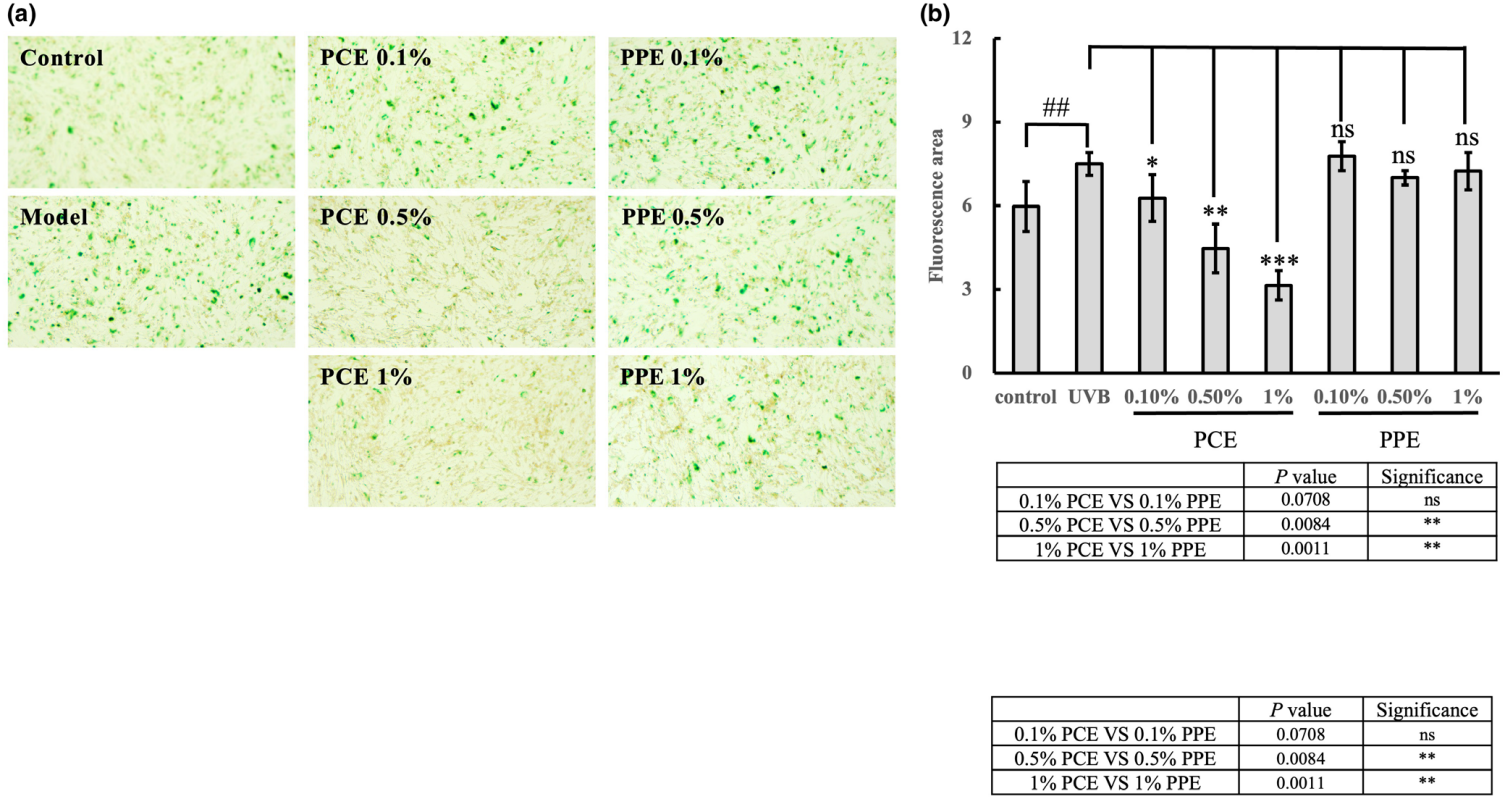
The effect of PCE on UVB stimulated senescent cells by β‐galactosidase staining. (a) The microscopy images of senescent cells by β‐galactosidase staining in HSF cells. (b) The statistical results of the senescent cells. One‐way analysis of variance (ANOVA) was performed under UVB condition. *p < 0.05; **p < 0.01; ***p < 0.001 (*n* = 3); n.s. means no significance. The Student's *t*‐test was compared between control and UVB treatment. ##*p* < 0.01. In the table below, the Student's *t*‐test was compared between PCE and PPE at the same concentration.

MMPs are a group of enzymes that break down collagen and elastin in the skin. When the skin is exposed to UVB radiation, the skin cells could be stimulated to produce more MMPs. When more MMPs are produced, the skin ageing process is accelerated. We performed quantitative Reverse Transcription Polymerase Chain Reaction (qRTPCR) to test the gene expression of *MMPs* in UVB‐induced fibroblasts. As opposed to blank control, the exposure to UVB significantly induced the gene expressions of *MMP1*, *MMP3* and *MMP9* by 305.3%, 55.5% and 68.5%, respectively (*p* < 0.05; Figure 4). Notably, even at a low concentration (0.1%), PCE showed significant suppression of *MMP1* (10.6% reduction; *p* < 0.05; Figure 4a) and *MMP9* (44.3% reduction; *p* < 0.001; Figure 4c). The results suggested that PCE exhibited an inhibition effect on *MMP1*, *MMP3* and *MMP9* gene expressions against UVB (Figure 4).

### PCE decreased the number of senescent cells

**FIGURE 5:**
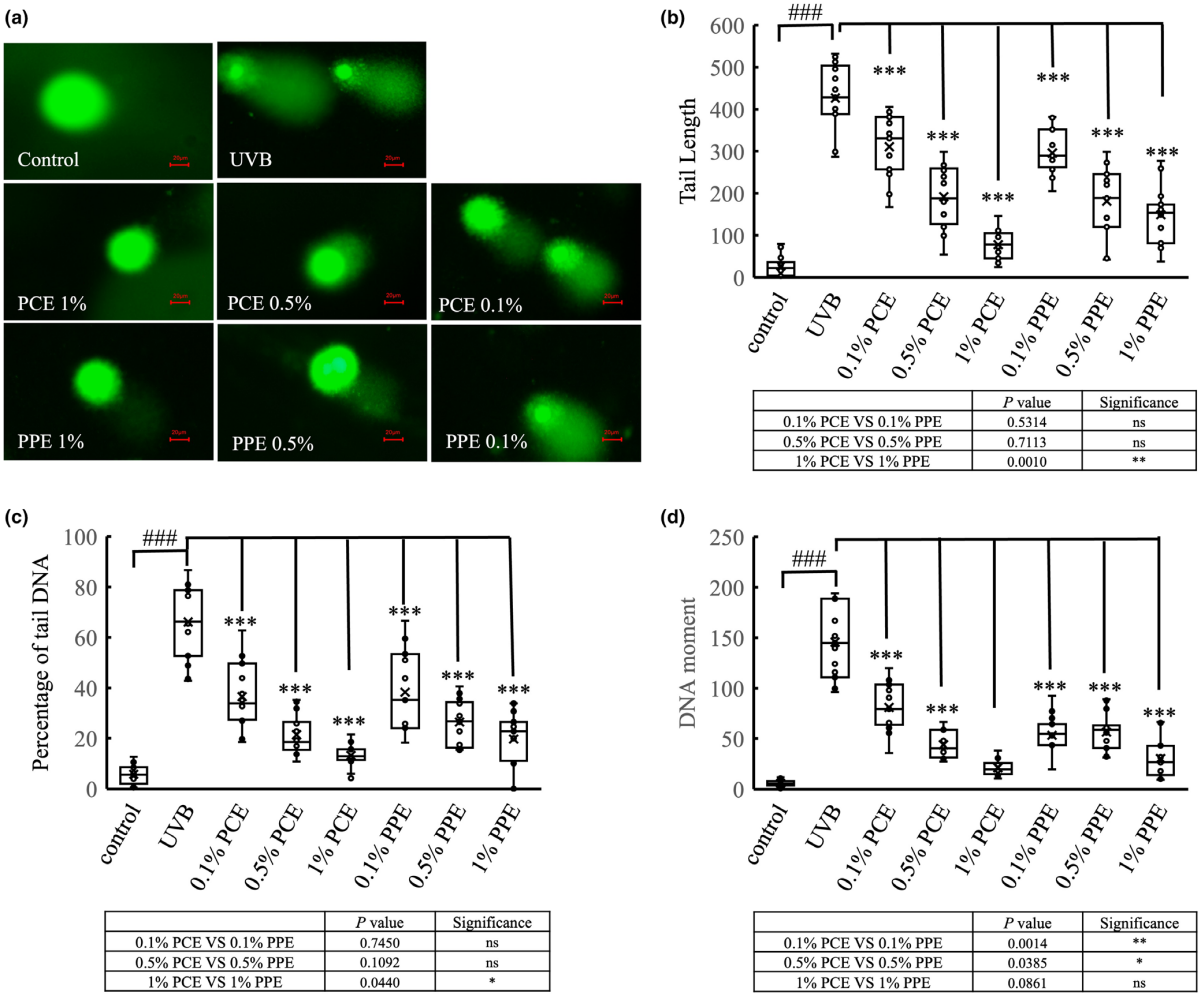
The effect of PCE on DNA damage in UVB‐stimulated HSF cells by comet assay. (a) The fluorescence images of comet assay in HSF cells. The scale bar represents 20 μm. The DNA damage is total percentage of DNA in the tail length (b), percentage of tail DNA (c) and DNA moment (d). One‐way analysis of variance (ANOVA) was performed under UVB condition. *p < 0.05; **p < 0.01; ***p < 0.001 (*n* = 12). The Student's *t*‐test was compared between control and model group (UVB exposure, C48/80 treatment). ### Represents *p* < 0.001 (*n* = 12). In the table below, the Student's *t*‐test was compared between PCE and PPE at the same concentration.

We assessed the production of a marker of cellular senescence—the activity of β‐galactosidase in fibroblasts (Figure 5a)—to evaluate the role of PCE in anti‐ageing. Over a concentration of 0.5%, the addition of PCE resulted in a notable reduction in the activity of β‐galactosidase. Specifically, 0.1% and 0.5% of PCE decreased 16.4% (*p* < 0.05) and 59.3% (*p* < 0.01) of β‐galactosidase activity respectively. While after the treatment of 1% of PCE, the activity of β‐galactosidase significantly decreased by 58.7% (*p* < 0.001; Figure 5b). The results in Figure 5b suggest that both 0.5% and 1% of PCE exhibit an inhibition on the ageing process of UVB‐induced fibroblasts. The inhibition is much stronger than PPE at the same concentration.

### PCE promoted the collagen content in vivo

**FIGURE 6:**
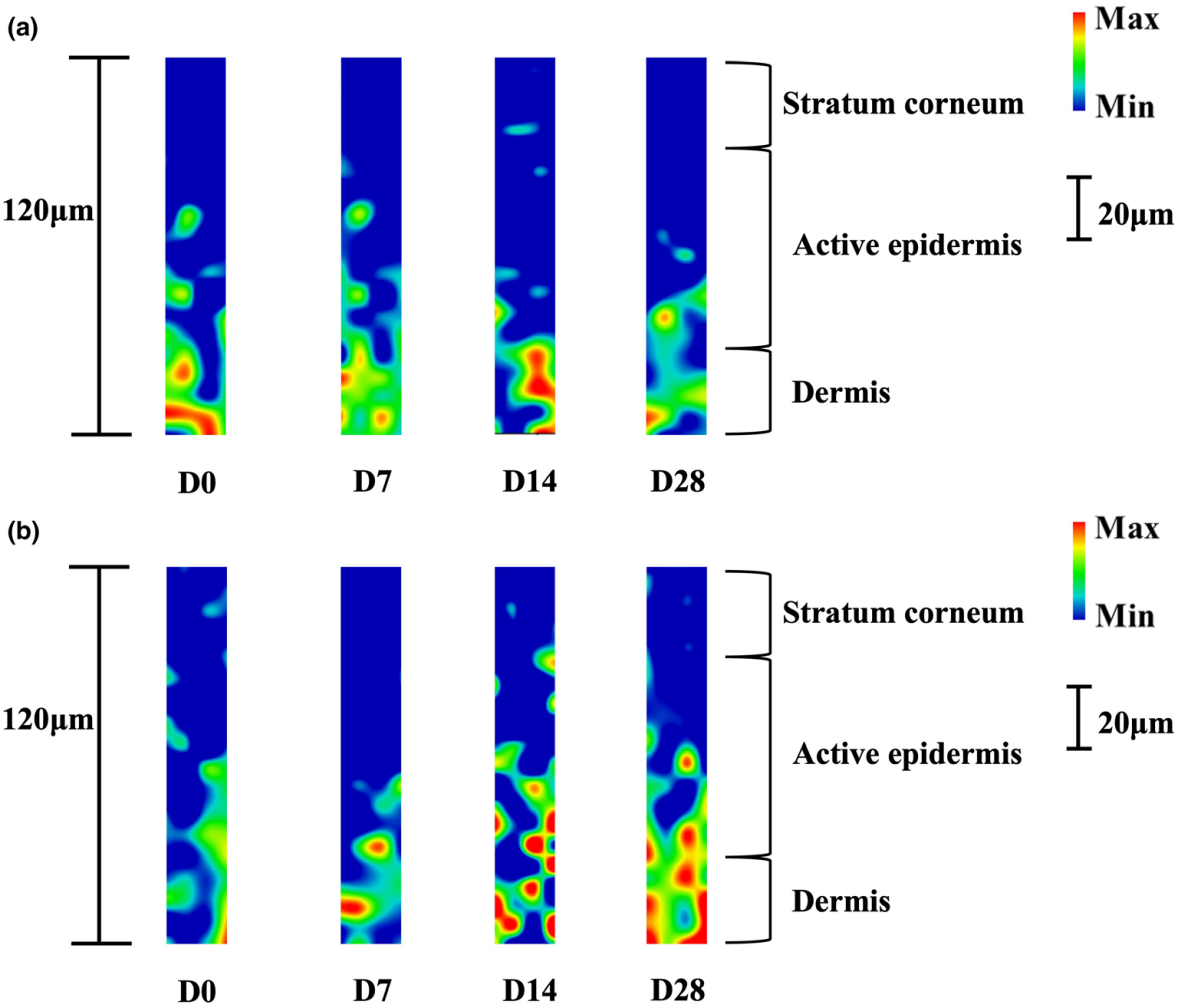
Raman detection of the endogenous collagen content in vivo after application of the cosmetic formulation without PCE (A) and formulation containing 1% PCE.

To assess the anti‐ageing effects of skincare products containing 1% PCE, we utilized Raman confocal microscopy to monitor the changes in endogenous collagen content after 7, 14 and 28 days of application. The image in Figure 6 clearly shows the distribution and content of total endogenous collagen in the skin of the subjects at different time points. When using the formulation without PCE, compared to Day 0, the skin's total collagen content increased by 0.36%, 1.21% and 0.52% after 7, 14 and 28 days, respectively (Figure 6a). However, using the formulation containing 1% PCE, the total collagen content increased by 5.58% at 7 days, 7.11% at 14 days and 9.35% at 28 days, respectively, compared to Day 0 (Figure 6b). In general, these results show that PCE could enhance the content of collagen proteins in the skin.

## MATERIALS AND METHODS

### Peony callus induction, callus culture and extract

After disinfection using 70% ethanol and 10% sodium hypochlorite, the fresh peony buds were cut into 10 mm pieces with a sterilized scalpel and made on Murashige and Skoog (MS) medium, containing 30 g L^−1^ sucrose, 7 g L^−1^ agar, 0.5 mg L^−1^ 6‐benzylaminopurine (6‐BA), 2 mg L^−1^ thidiazuron (TDZ) and 0.5 mg L^−1^ α‐naphtaleneacetic acid (NAA). The segments were incubated in the growth chambers at 25 ± 1°C under dark conditions for 20–30 days to induce callus. After the induction, for the proliferation, the callus was transferred to another MS medium. After 25 days of proliferation, the callus was collected and then frozen in a vacuum freeze dryer (Songyuan Huaxing Technology Develop Co., Ltd., Beijing, China) for 48 h to obtain the dried callus.

The dried callus was ground into fine powder in liquid nitrogen dissolved with 50% ethanol at a material‐to‐liquid ratio of 1:10 and then extracted with the aid of ultrasound (2000 W) for 90 min. Subsequently, the mixture was centrifuged at 15 000 rpm for 10 min and filtered using a 0.22 μm filter (Jinteng Technology Co., Ltd., Tianjin, China) to obtain the supernatant. After rotary evaporation of excess solvent, the final extract (PCE) was then used in this study. By the same extraction method, whole PPE was obtained for comparison.

### Cell culture and treatments

HaCaT, human skin fibroblasts cell line and human mast cells were obtained from Qingqi Biotechnology Development Co., Ltd. (Shanghai, China). HaCaT, fibroblasts and mast cells were cultured in Dulbecco's modified Eagle's medium (DMEM) containing 10%–15% foetal bovine serum (FBS), 100 U/mL penicillin and 100 μg/mL streptomycin, in a humidified 5% CO_2_ incubator at 37°C.

Before application, the solution of PCE or PPE was diluted in DEME to obtain the final concentration of 0.1%, 0.5% and 1% (v/v) respectively. To study the alleviation effects of PCE on UVB stress, 30 mJ/cm^2^ UVB was used to establish the model. Unless otherwise specified, model refers to UVB treatment. Firstly, the sample groups and the model group were stimulated with UVB (30 mJ/cm^2^). After radiation, PCE or PPE at different concentrations was added to the cell culture plate, and incubated at 5% CO_2_, 37°C overnight. In parallel, the blank control and the model group were added serum‐free DMEM medium.

### Cell viability measurement

HaCaT and fibroblasts of logarithmic growth stage were taken and inoculated on 96‐well plates by 9 × 10^3^ cells/well, incubated in an incubator at 37°C and 5% CO_2_ for 24 h and then the superserum was discarded and cleaned twice with PBS. The experimental samples were dissolved in the culture medium containing fresh DMEM and arranged into five concentration gradient solutions, 100 μL per well, and incubated for 24 h. After adding 10 μL CCK‐8 reagent to culture for 2 h, OD value at 450 nm was measured.

$$\text{Cell viability}\;\left(\%\right)=\left(\mathrm{OD}\;\text{of treatment group}-\mathrm{OD}\;\text{of control group}\right)/\left(\mathrm{OD}\;\text{of control}\right)\times 100.$$

### qRT‐PCR

HaCaT and fibroblasts in the logarithmic growth phase were seeded into a six‐well cell culture plate at a density of 1 × 10^5^/well. After the cells adhered overnight, the treatment group and model group were exposed to UVB. Then, PCE was added to the cell culture plate and incubated for 24 h. Simultaneously, the blank control was set up using serum‐free DMEM medium without UVB radiation. Subsequently, the gene expression levels of *TRPV1*, *IL‐1α* and *IL‐6* in HaCaT cells, meanwhile *MMP1*, *MMP3* and *MMP9* in fibroblasts were measured.

Total RNA was isolated from cells using an EasyPure® RNA Kit (TransGen Biotech, Beijing, China), and cDNA was synthesized using a TransScript® One‐Step gDNA Removal and cDNA Synthesis SuperMix (TransGen Biotech, Beijing, China). Quantitative real‐time PCR was performed in the QuantStudio 5 Real‐Time PCR System (Thermo Fisher Scientific Inc., Massachusetts City, USA) with a 20 μL reaction volume containing 1 μL of cDNA, 7 μL ultrapure water, 1 μL forward primer, 1 μL reverse primer and 10 μL Thunderbird SYBR qPCR Mix (Toyobo Co., Ltd., Ōsaka shi, Japan). The thermocycler parameters were 95°C for 30 s, followed by 40 cycles of 95°C for 10 s and 60°C for 30 s. To analyse the relative expression levels of target genes, the comparative CT (2^−ΔΔCT^) method was utilized. The sequences of primers used in this study are listed in Table 1. β‐Actin was used as the control gene for normalization.

**TABLE 1:** Primer sequence of the target genes.

Gene	Sequence
*MMP1*	F: 5′AAAATTACACGCCAGATTTGCC 3′
	R: 5′ GGTGTGACATTACTCCAGAGTTG 3′
*MMP3*	F: 5′ CTGGACTCCGACACTCTGGA 3′
	R: 5′ CAGGAAAGGTTCTGAAGTGACC 3′
*MMP9*	F: 5′ TGTACCGCTATGGTTACACTCG 3′
	R: 5′ GGCAGGGACAGTTGCTTCT 3′
*IL‐1α*	F: 5′ TGGTAGTAGCAACCAACGGGA 3′
	R: 5′ ACTTTGATTGAGGGCGTCATTC 3′
*IL‐6*	F: 5′ ACTCACCTCTTCAGAACGAATTG 3′
	R: 5′ CCATCTTTGGAAGGTTCAGGTTG 3′
*TRPV1*	F: 5′ AGCACCTCACAGACAACGAG 3′
	R: 5′ ATGGTGGTGTTCTGTCCGTC 3′
*β‐Actin*	F: 5′ CGGGAAATCGTGCGTGAC 3′
	R: 5′ GGAAGGAAGGCTGGAAGAGTG 3′

### Measurement of histamine contents

To assess the effect of PCE on prompt alleviation, the release of histamine from mast cells was detected, according to Koibuchi et al. [21]. 1 × 10^6^ mast cells were seeded in six‐well plates until the cell fusion reached 70%–80%. After that, the cells were changed medium supplied with 5 μg/mL C48/80 (Sigma‐Aldrich, Missouri, USA) and incubated at 37°C for 1 h. Afterwards, 0.1%, 0.5% and 1% of PCE were added in cell plates, except blank control. After additional incubation for 24 h, conditional supernatant was harvested for assay using centrifugation at 1500 rpm for 15 min. The concentration of histamine was determined using an enzyme‐linked immunosorbent assay kit (Jiancheng Bioengineering Institute, Nanjing, China). Samples of supernatant and histamine standards (50 μL each) were added to the wells of a coated microplate, followed by the addition of 50 μL of biotin‐labelled antigen working solution. The plate was incubated at 37°C for 30 min. After incubation, the wells were washed five times, then 50 μL of streptavidin‐HRP working solution was added to each well and the plate was again incubated at 37°C for 30 min. The wells were washed five times again, 50 μL of substrate solution A and 50 μL of substrate solution B were added to each well, followed by incubation at 37°C for 10 min. Finally, 50 μL of stop solution was added to each well. The optical density (OD) was measured at 450 nm using a microplate reader (Varioskan LUX, Thermo Scientific, USA), and the concentration of histamine was calculated based on the standard curve.

### Comet assay for DNA damage

Fibroblasts were plated in six‐well plates 1 day before UVB (30 mJ/cm^2^) exposure. After exposure, the cells in the sample group were cultured with PCE for 24 h. Afterwards, the cells were centrifuged at 1000 rpm for 5 min to collect cell pellets. The comet assay was performed using the comet assay kit (Cell Biolabs, San Diego, USA). Briefly, the cells were lysed, and electrophoresis was conducted under alkaline conditions. Electrophoresis was carried out with a voltage of 25 V and a current of 300 mA for 25 min. The electrophoresis tank was maintained at 4°C. Then, 50 μL of Hoechst stain was applied to each gel and the samples were incubated in the dark for 10 min. The slides were then examined under a fluorescence microscope (ICX41, SOPTOP, China) at 200× magnification. The results were analysed using ImageJ software (National Institute of Health, Bethesda, MD, USA). A total of 30 cells per sample were selected to determine the average percentage of DNA damage.

### Senescence‐associated β‐galactosidase staining assay

The population of senescent cells was determined by assessing the cellular β‐galactosidase activity using a senescence β‐galactosidase staining kit (Beyotime Biotechnology, Shanghai, China). Briefly, fibroblasts were seeded in six‐well plates at a density of 1 × 105 cells/well and cultured overnight. Then, the cells were treated with DMEM culture medium containing 0.1%, 0.5% and 1% PCE for 48 h respectively. The cells were then fixed with fixative solution for 15 min at room temperature, washed with PBS three times after the fixation and then incubated with 1 mL of β‐galactosidase staining solution at 37°C overnight. β‐Galactosidase‐positive (blue staining) cells were counted in 10 random fields for each well under a phase‐contrast microscope (magnification at 100×). The average of β‐galactosidase‐positive cells for each field of view was calculated. The experiment was repeated three times for each treatment.

### Collagen content measured in the skin using Raman confocal spectroscopy

Two groups of 13 healthy humans (age range 25–35 years old) were recruited and signed the informed consent form before Raman test. One group used the cosmetics formulation (water 98.1%, hydroxyacetophenone 0.4% and 1,2‐hexanediol 0.5%) containing 1% PCE, and the other group applied the same formulation without PCE. The cosmetics were applied twice daily on the inner side of the forearm by the volunteers. The measurements were performed at the marked area on Day 0, Day 7, Day 14 and Day 28.

Raman confocal microscope system (LabOdessy, Horiba, Japan) equipped with a 532 nm (He‐Ne laser) laser source, a semiconductor‐cooled CCD detector (1024 × 800 pixels) and a 50 μm confocal pinhole and 50 objective (NA 0.50, 10.6 mm) was used in this study. A standard single‐point calibration was performed using a silicon line (520.5 cm^−1^). The laser power was set at 0.02 mW, and the integration time of each point was 0.05 sec. Data were acquired in the spectral range of 400–4000 cm^−1^ with a spectral resolution of 2.8 cm^−1^. Spectral images were acquired in a point‐by‐point mode on an X‐Y‐Z platform. X‐axis (parallel to the skin surface) steps were 10 μm and Y‐axis (perpendicular to the skin surface) steps were 10 μm. To compare the intensities of the bands in the region of 400–4000 cm^−1^, the spectra were normalized using 1004 cm^−1^ band relative to phenylalanine, and a smoothing procedure was used. The clinical test was approved by the Ethics Committee of Shanghai WEIPU Testing Technology Group Co., Ltd. on 2 February 2023 (ethics review approval document number: WP‐202302JC04).

### Statistical analysis

Results were displayed as mean ± SD. Statistical analysis was performed using one‐way analysis of variance (ANOVA) to compare the PCE‐treated groups, with GraphPad Prism 6.0 (GraphPad Software, Inc., San Diego, USA). The Student's *t*‐test was performed between control and model group (UVB exposure). */#, represents *p* < 0.05, **/## represents *p* < 0.01 and ***/### represents *p* < 0.001.

## DISCUSSION

Many consumers value the use of safe products, therefore, they prefer cosmetics made with natural ingredients, particularly botanical sources [22, 23]. However, there is need for substantial quantities of natural plant material to create plant extracts using conventional ways. Therefore, the demand births challenges to environmental protection and ecological network stability. In addition, botanical sources vary because of diverse origins, climates and growth conditions [24, 25]. In recent years, the food, cosmetics and pharmaceutical industries mostly use plant callus tissue. The tissue is known for its various benefits including continuous production, reliable quality, land‐saving features, no limitations of whether, no contamination and good sustainability [26, 27, 28, 29]. This study aimed to establish stable and successive generations of plant callus cells via plant tissue culture techniques (Figure 1a). We used the buds of the rare ‘Peony King’ as explants to achieve the goal of introducing the new cells. From the cell viability assessments, our results showed negligible cytotoxicity in PCE. These findings suggest that PCE is a safe ingredient for cosmetic formulations (Figure 1c).

UV radiation could penetrate the epidermis and trigger a series of reactions in molecular events. The events lead to inflammatory responses and compromise the function of skin as a barrier [30]. Mast cells are one of the primary immune cells in skin. When mast cell receptors detect damage signals, they trigger degranulation, which results in the rapid release of histamine and other inflammatory mediators. The release of histamine increases vascular permeability and elicits symptoms such as redness, swelling and itching [31]. Shinoda et al. [32] discovered that histamine could enhance the release of IL‐6. The pro‐inflammatory cytokines, such as IL‐1α and IL‐6 in response to UVB exposure, contribute to the initiation and propagation of cutaneous inflammation, exacerbating tissue damage and causing skin sensitivity [11, 33]. Thus, a strategy aimed at mitigating UVB‐induced damage may include using cosmetic formulations containing reagents that reduce the inflammatory series of reactions and enhance the integrity of the skin barrier. Our results indicated that PCE had an anti‐inflammatory effect by decreasing the gene expressions of *IL‐1α* and *IL‐6* (Figure 2). TRPV1 expresses widely in skin sensory nerve fibres, including Aδ and C‐fibres, and regulates the influx of Ca^2+^ [34, 35, 36]. Activation of TRPV1 on sensory neurons elicits sensations of pain, heat and itching [37, 38]. Here, we found a potential soothing extract derived from peony callus tissue. The active ingredient in PCE could soothe the skin by decreasing the expressions of inflammation factors, TRPV1 and histamine induced by UVB (Figure 2). We found paeoniflorin in PCE (Figure S1). Our finding was consistent with the result that *Paeonia lactiflora Pall* extract containing peony glucosides (mainly paeoniflorin) had a pharmacological effect of anti‐inflammation [1].

Our findings indicate that, in addition to soothing properties, PCE has remarkable anti‐ageing effects. Ageing skin commonly presents with features including diminished elasticity, the formation of wrinkles, reduced luminosity, the emergence of hyperpigmented lesions and symptoms of pruritus [39]. Numerous active compounds and botanical extracts exhibiting anti‐ageing efficacies have been reported, including *Leontopodium alpinum* [40, 41] and *Citrus junos* [42]. Our results suggested that PCE exhibited inhibition on collagen degradation, primarily attributed to the suppression of MMP activity, notably MMP1, MMP3 and MMP9 (Figure 4). It is consistent with previous studies that UVB induced by the increasing of MMP1 which led to collagen degradation could be suppressed by peony extract [43]. Furthermore, its beneficial anti‐ageing properties were assessed through β‐galactosidase activity assay (Figure 5). Emerging evidence indicates that DNA damage is a likely initiator of cell senescence [44]. The alkaline comet assay, renowned for its sensitivity, enables the detection of DNA strand breaks and alkali‐labile sites across diverse eukaryotic cell types [45, 46]. We performed the alkaline comet assay to assess UVB‐induced DNA damage and evaluate the protection of PCE against such damage. Our results revealed a substantial induction of DNA damage by UVB radiation. However, the addition of PCE, even at a concentration as low as 0.01%, markedly mitigated UVB‐induced DNA damage (Figure 3). To identify the role of PCE in anti‐ageing, we subsequently detected the changes in intrinsic collagen contents in vivo using Raman spectroscopy, which has been regarded as an advanced method for monitoring the collagen content within dermis in vivo [47, 48]. Following continuous use of a cosmetic formulation containing 1% PCE for 28 days, in contrast with formulation without PCE, there was a sustained increase in collagen protein content (Figure 6). Collectively, these findings suggest that PCE shows eminent anti‐photoageing properties, which is effective in enhancing the level of collagen and reducing collagen degradation.

In summary, the utilization of PCE represents a safe and promising application in the realm of cosmetic formulations. PCEs have eminent soothing properties and demonstrated anti‐ageing attributes. The soothing properties of the extracts make them compelling for use in skincare products. PCE could soothe skin inflammation and enhance resilience against ageing factors. The superior function of the extracts highlights its potential as a key ingredient in cosmetic formulations designed to promote skin health and vitality. Moreover, most manufacturers prefer using PCE as the prime choice because it is natural and safe to use cosmetic formulations. Using peony callus tissue over traditional peony plants is a sustainable way of using resources. From the study, we conclude that PCE is more effective in reducing histamine release and decreasing the number of senescent cells compared to PPE. These results indicate that PCE has significant potential for practical use in skincare products, meeting the demands of consumers and providing effective and safe skincare solutions.

## CONFLICT OF INTEREST STATEMENT

The authors declare no conflict of interest.

## Supporting information


Data S1.

## Data Availability

Data sharing is not applicable to this article as no datasets were generated or analysed during the current study.
