# Supplementary material for: Protective effects of Paeonia suffruticosa callus extract in skin through anti‐inflammation and repair UVB‐induced damage
Source: Int J Cosmet Sci. 2025 Mar 13;47(4):652–64. doi: 10.1111/ics.13055 (PMC12319501; doi:10.1111/ics.13055)
Supplement: Supplementary file 1 — Data S1. [file ICS-47-652-s001.docx]

**Supplementary Information**

**Supplementary Methods**

**HPLC identification of Peony callus extracts**

To evaluate the main components, paeoniflorin and paeonol in PCE, we conducted the qualitative analysis using high-performance liquid chromatography (HPLC). 5 mg of paeoniflorin and paeonol standard sample (Sigma) were weighed, dissolved in methanol to obtain the standard stock solution of 1 mg mL^-1^. The stock solutions were diluted with methanol to the final concentrations of 0.1 mg mL^-1^, 0.2 mg mL^-1^, 0.3 mg mL^-1^, 0.4 mg mL^-1^and 0.5 mg mL^-1^. The standard curve was drawn, and the regression equation was calculated. The PCE was passed through 0.22 μm organic filter membrane before testing, and 1.5 mL of PCE was taken as the test sample.

HPLC analysis was performed on Vanquish Core (Thermo, USA), equipped with DAD Detector (VC-D40-A-01, Vanquish™ Tunable Wavelength, Thermo, USA). A Shim-pack GIST C18 column (250 × 4.6 mm; 5 μm; Shimadzu, Japan) was used. The column temperature was maintained at 25°C, the flow rate was 1 mL min^-1^ at a detection wavelength of 230 nm (He et al., 2013). Binary gradient elution was adopted, mobile phase A was water (0.05% TFA), B was acetonitrile, elution procedure: 0~5 min, 0%~5% acetonitrile; 6~20 min, 5%~20% acetonitrile; 21~50 min, 25%~50% acetonitrile. All reagents, including acetonitrile, methanol, and water, were of HPLC grade. All solutions were filtered through ultrasonic filtration prior to use. The area ratios of the absorption peaks were calculated.

**Supplmentary Figure 1. The main ingredient paeoniflorin and paeonol in PCE by HPLC detection.**


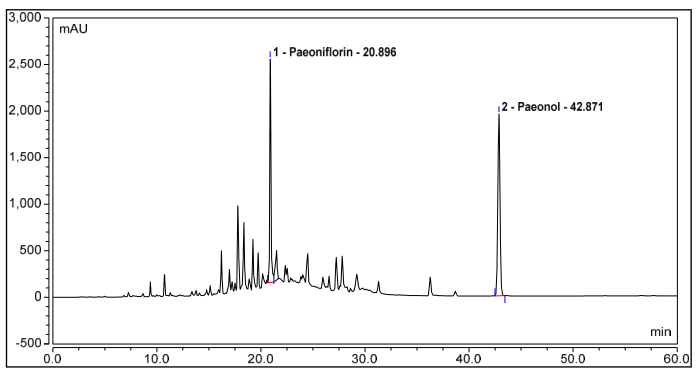


**Supplementary References**

He, C., Peng, Y., Xiao, W., Liu, H., Xiao, P. G. Determination of chemical variability of phenolic and monoterpene glycosides in the seeds of Paeonia species using HPLC and profiling analysis. Food Chemistry, 2013, 138, 2108-2114.
